# Coronary artery fistula following surgical myectomy for hypertrophic obstructive cardiomyopathy: a case report

**DOI:** 10.1093/ehjcr/ytae248

**Published:** 2024-05-16

**Authors:** James Bowles, Joshua Martin, Penni L Russell, Amy Bailey, David J Holland

**Affiliations:** Department of Cardiology, Sunshine Coast University Hospital, 6 Doherty Street, Birtinya, 4575 Queensland, Australia; Department of Cardiology, Sunshine Coast University Hospital, 6 Doherty Street, Birtinya, 4575 Queensland, Australia; Department of Cardiology, Sunshine Coast University Hospital, 6 Doherty Street, Birtinya, 4575 Queensland, Australia; Department of Cardiology, Sunshine Coast University Hospital, 6 Doherty Street, Birtinya, 4575 Queensland, Australia; Department of Cardiology, Sunshine Coast University Hospital, 6 Doherty Street, Birtinya, 4575 Queensland, Australia; School of Medicine and Dentistry, Griffith University, 6 Doherty Street, Birtinya, 4575 Queensland, Australia; School of Human Movement and Nutrition Sciences, The University of Queensland, Brisbane, 4072 Queensland, Australia

**Keywords:** Coronary artery, Fistula, Echocardiography, Hypertrophic cardiomyopathy, Myectomy, Case report

## Abstract

**Background:**

Coronary artery fistula is a rare, but recognized complication of surgical myectomy. Although most communicate with the right heart, a large fistula into the left ventricular cavity may result in a shunt haemodynamically analogous to aortic regurgitation. Understanding the variable presentation of iatrogenic coronary fistulae and the optimal evaluation strategy is critical to obtaining a timely diagnosis and instituting treatment.

**Case summary:**

We report the case of a 57-year-old renal transplant recipient admitted for evaluation of presyncope, one-year post-surgical myectomy for hypertrophic obstructive cardiomyopathy. An iatrogenic coronary artery fistula was suspected by transthoracic echocardiography, and later confirmed with both non-invasive and invasive coronary angiography.

**Discussion:**

We highlight various cardiac imaging modalities that confirmed the diagnosis of coronary artery fistula and helped to determine the clinical significance. We report the tailored approach often required to determine the anatomic and haemodynamic characteristics of coronary fistulae and outline potential management strategies.

**Learning points**

- Though most coronary artery fistulae are congenital, iatrogenic fistulae may result from cardiac procedures such as septal myectomy.
- The appearance of a coronary artery fistula on echocardiography depends on the anatomical/shunt characteristics and timing of imaging. Coronary angiography should be considered for anatomical confirmation.
- Multimodality imaging is not only helpful in detecting coronary fistulae but also for determining the clinical and haemodynamic significance, and for strategic planning when intervention is required.

## Introduction

Septal reduction therapy is a guideline-endorsed treatment option for patients with hypertrophic obstructive cardiomyopathy (HOCM) and symptoms refractory to optimal medical therapy.^1,2^ We present a case report of an infrequently encountered complication of surgical myectomy—a coronary artery fistula (CAF) between the first septal perforator of the left anterior descending artery and the left ventricular (LV) cavity, and highlight its variable appearance on multimodality cardiac imaging.

## Summary figure

Coronary artery fistula of the septal perforator on echocardiography and cardiac computed tomography. Pre-surgical angiogram demonstrating normal course of the septal perforator with post-surgical angiogram demonstrating abrupt termination of the first septal vessel with contrast blush present within the left ventricular cavity.

**Figure:**
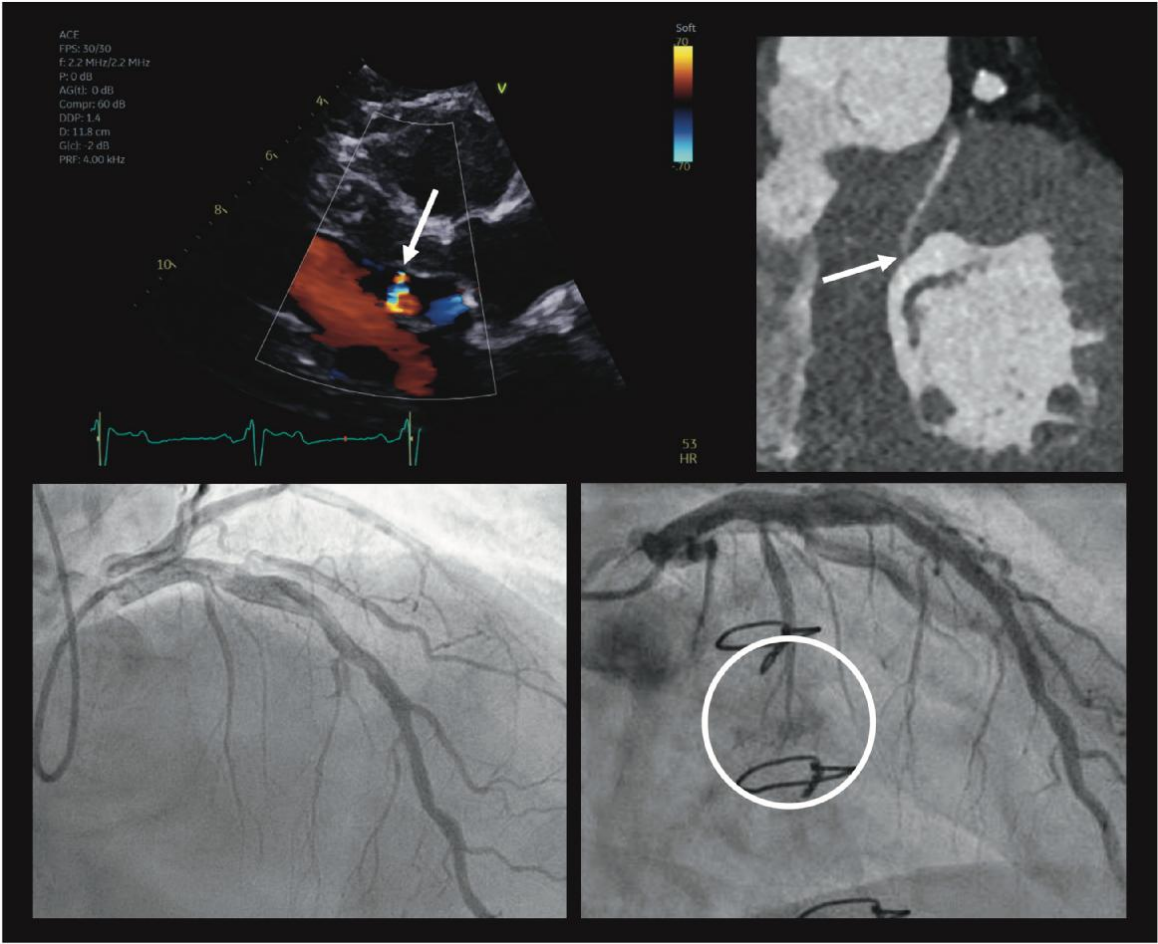


## Case report

A 57-year-old male renal transplant recipient was admitted to hospital for evaluation of recurrent presyncope. He had a previous history of surgical myectomy for HOCM, renal transplant for end-stage renal failure secondary to reflux nephropathy, hypertension, and dyslipidaemia. Relevant medications included metoprolol 25 mg mane and 50 mg nocte, atorvastatin 20 mg, tacrolimus 3 mg, mycophenolate 500 mg twice daily, prednisolone 5 mg daily, and trimethoprim-sulfamethoxazole 80/400 mg daily. On examination, heart rate was 63 b.p.m. and blood pressure 106/70 mmHg, with evidence of orthostatic hypotension (standing blood pressure 86/57 mmHg). There was a loud systolic murmur (but no diastolic murmur) heard at the left sternal edge, which radiated to the carotid arteries. There was no significant augmentation with dynamic manoeuvres, nor were there clinical signs of heart failure.

**Figure 1:**
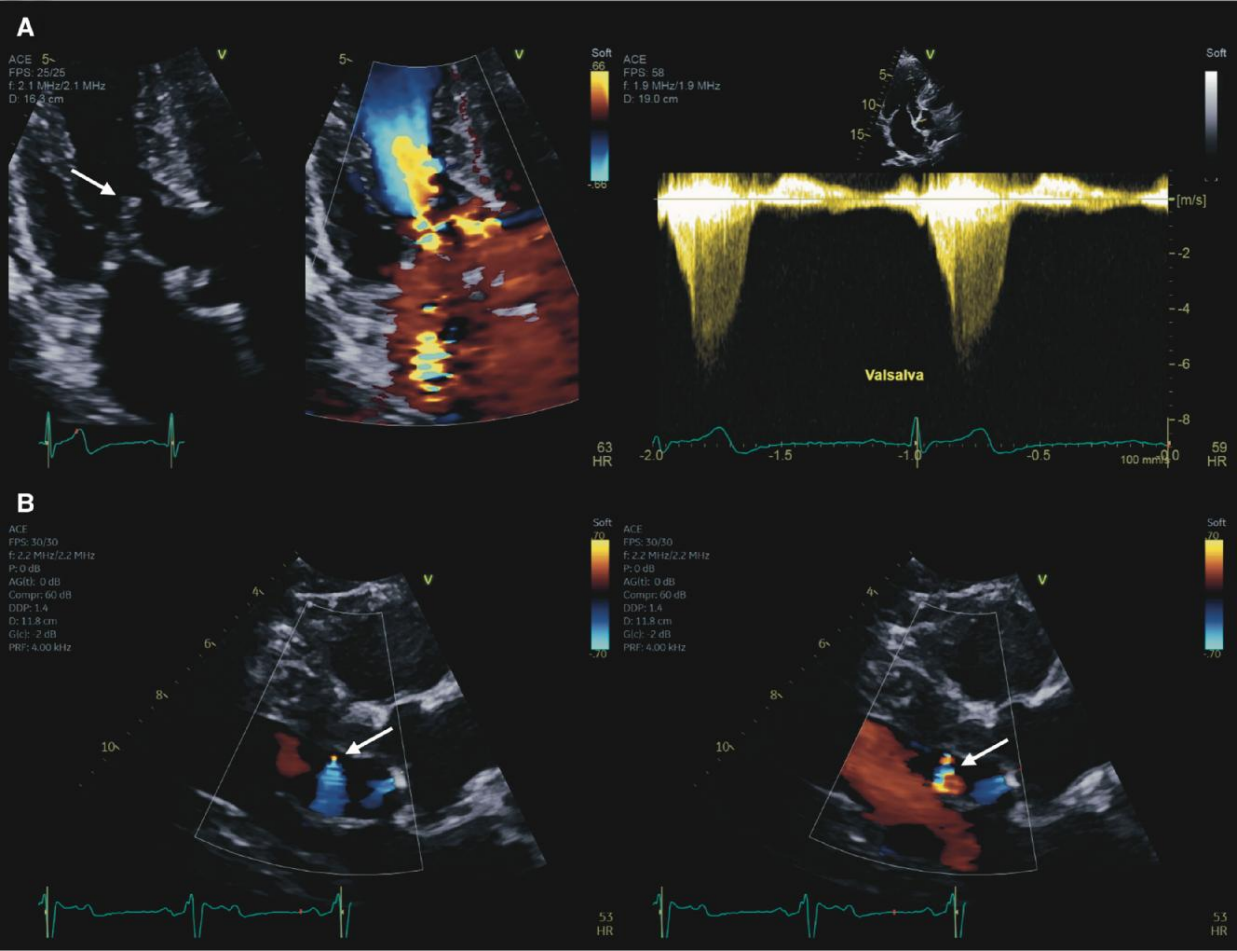
(*A*) Pre-surgical echocardiography demonstrating septal hypertrophy and dynamic left ventricular outflow obstruction (right upper panel) due to systolic anterior motion of the mitral valve (left upper panel, arrow). (*B*) Post-surgical colour Doppler demonstrating flow from the ventricular septum to the left ventricular cavity (arrows). Additional echocardiography imaging is available in the Supplementary material online.

One year earlier, the patient had undergone surgical myectomy for HOCM with persistent class II–III dyspnoea, and presyncope on medical therapy. Despite appropriate resting bradycardia, the pre-myectomy resting echocardiogram had demonstrated severe dynamic left ventricular outflow tract (LVOT) obstruction (85 mmHg at rest and 140 mmHg on Valsalva manoeuvre) associated with increased septal thickness (18 mm) and significant mitral valve systolic anterior motion resulting in moderate-to-severe mitral regurgitation (*Figure 1A*). Left and right ventricular volumes and ejection fraction (EF 55%) were normal. Pre-myectomy exercise stress echocardiography was electrically and symptomatically negative for inducible ischaemia (at 9.4 METs, 83% maximum predicted heart rate), but showed evidence of severe dynamic intracavity obstruction post-exercise and moderate-severe dynamic mitral regurgitation despite continued beta-blocker therapy. Coronary angiography revealed a right-dominant coronary system with type II left anterior descending artery and non-obstructive coronary artery disease. Initial risk calculation for HOCM (2.5% by the European Society of Cardiology HCM Risk-SCD calculator^3^) did not recommend primary-prevention cardiac defibrillator implantation.

**Figure 2:**
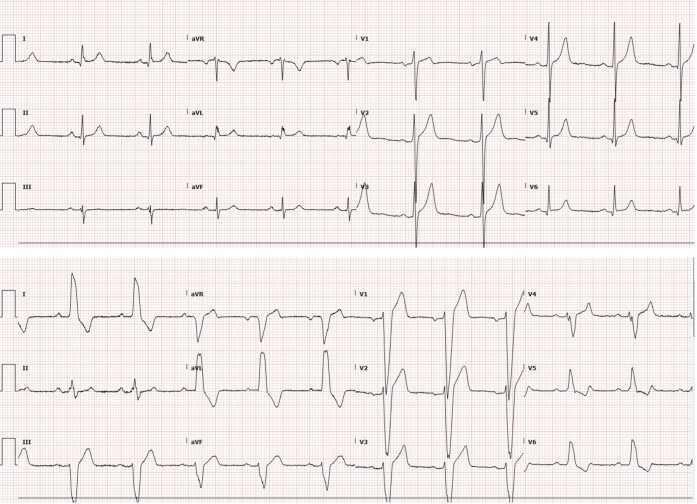
Electrocardiography demonstrating voltage criteria for left ventricular hypertrophy at baseline (top), and development of left bundle branch block following surgical myectomy (bottom). Sweep speed 25 mm/s.

Surgical myectomy reduced septal thickness to 15 mm, and resulting in an improved (though still elevated) resting peak LVOT pressure gradient of 52 mmHg. Six months following surgery, he reported progressive exertional dyspnoea and orthostatic presyncope. A new left bundle branch block was noted (*Figure 2*). Given the known residual post-surgical LVOT gradient and occasional postural hypotension, repeat resting and stress echocardiography was arranged. Resting echocardiography demonstrated a now significant reduction in LVOT gradient (now 5 mmHg, 6 months post-myectomy), but an apparent shunt across the ventricular septum on colour Doppler imaging was noted (*Figure 1B*). Although appearing confined to diastole, the jet was not easily otherwise characterised, and an iatrogenic ventricular septal defect could not be excluded. After achieving 7 METS (60% of maximum predicted heart rate), the exercise stress echocardiogram was terminated due to fatigue and dyspnoea and did not induce ischaemia, arrhythmia, or a significant LVOT gradient (13 mmHg). Post-exercise right ventricular systolic pressure was also satisfactory at 30 mmHg.

**Figure 3:**
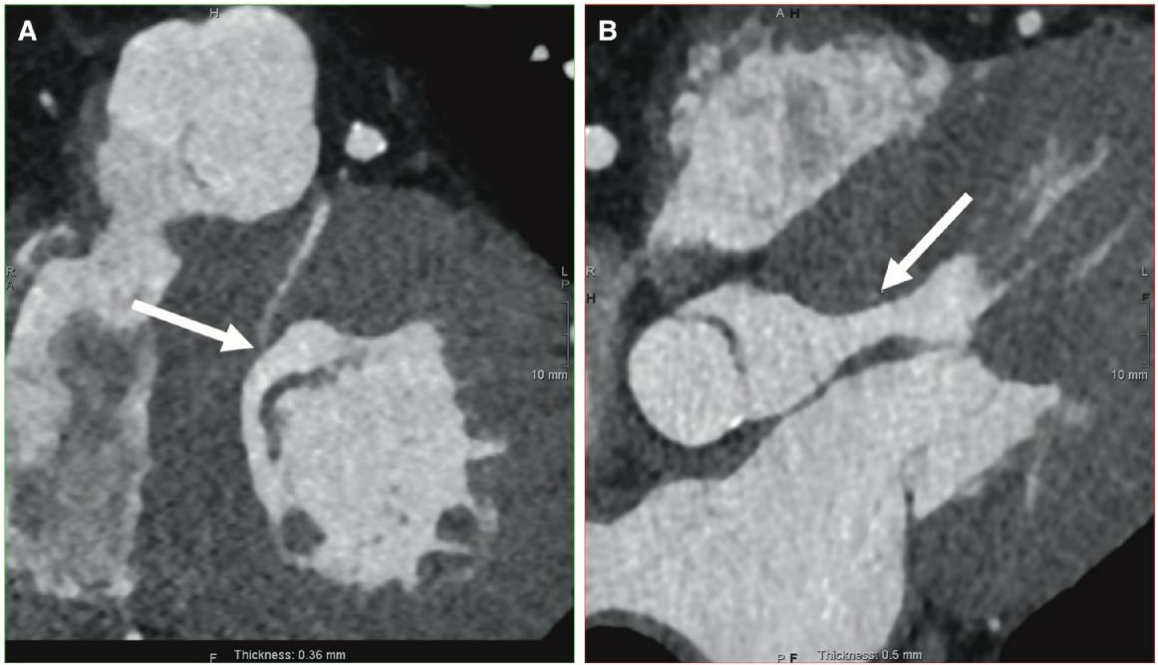
Computed tomography coronary angiography demonstrating the course of first septal perforator from the left anterior descending artery. Although the course of the perforator was determined by multiplanar reconstruction (*A*), its course into the left ventricular cavity could only be suggested (*B*).

**Figure 4:**
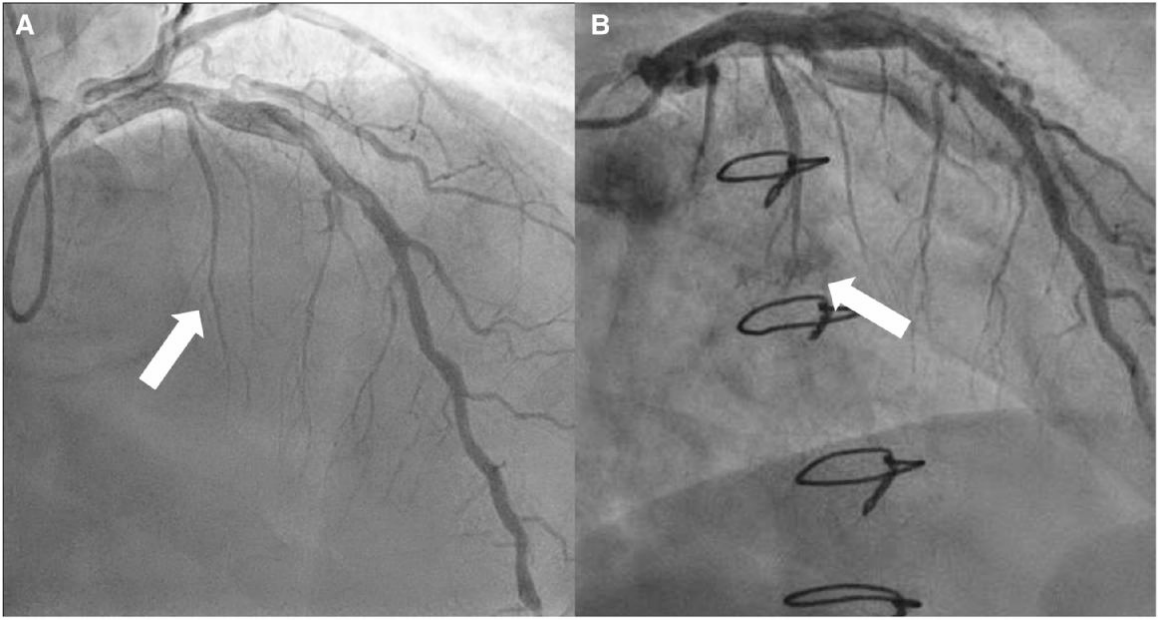
Coronary angiography performed for confirmation of coronary artery fistula. Pre-surgical angiogram demonstrating course of left anterior descending artery and first septal perforator (*A*). Post-surgical angiogram confirming communication of the first septal perforator with the left ventricular cavity (abrupt termination of the septal vessel with blush in the ventricular cavity, arrow *B*).

The patient proceeded to computed tomography coronary angiography (CTCA), which demonstrated a large calibre first septal perforating artery extending to the endocardial border of the LVOT. There was probable communication with the LV cavity (*Figure 3*), and a ventricular septal defect was excluded. Invasive angiography was performed to confirm the presence of a CAF, confirming a fistula between an unroofed first septal perforator and the LV cavity—an uncommon, but recognised finding following surgical myectomy (*Figure 4* and Supplementary material online). Given persisting symptoms and the left bundle branch block, which can complicate stress echocardiography, an adenosine myocardial perfusion scan was undertaken to confirm the absence of major ischaemia resulting from the CAF (see Supplementary material online). This demonstrated septal post-surgical wall motion changes and an EF of 59%, but there was no evidence of myocardial ischaemia to suggest a steal phenomenon. The CAF was, therefore, deemed appropriate for conservative management.

Inpatient telemetry demonstrated incidental runs of asymptomatic non-sustained ventricular tachycardia, prompting a recalculation of the HCM Risk-SCD score^3^ (6%). Accordingly, a primary-prevention defibrillator was subsequently implanted. Given the lack of symptom-rhythm correlation between ventricular tachycardia and presyncope, fludrocortisone was initiated for recurrent presyncope due to orthostatic hypotension, resulting in symptomatic improvement. Clinical stability has been confirmed on subsequent annual clinical assessments and 6-monthly device interrogation.

## Discussion and conclusion

We report the case of a CAF draining to the LV cavity post-surgical myectomy and highlight the role of multimodality imaging in the evaluation of such cases. A CAF occurs when there is an anomalous connection between one or more coronary arteries and an adjacent structure, either a cardiac cavity (i.e. coronary-cameral fistulae) or a pulmonary vessel (i.e. arterio-venous fistulae).^4–8^ Congenital causes account for most cases, whilst acquired causes including chest trauma, infection (i.e. bacterial endocarditis) and neoplasms account for the remainder.^4,6–9^ With the advent and widespread utilisation of interventional and surgical cardiac interventions, there is growing recognition of iatrogenic CAFs following valvular surgery, percutaneous or surgical coronary artery intervention, endomyocardial biopsy, and pacemaker insertion.^4,6–10^ Overall, CAFs are rare, occurring in 0.002–0.9% of the general population.^7,8^ They have been detected in 0.05–0.25% of invasive coronary angiogram series and 0.5% of dedicated CTCA series.^10^ Whilst exact incidence rates in post-cardiac interventional populations (e.g. post-surgical myectomy) are unknown, some case series authors suggest that they may be more common than thought, highlighted by one case series demonstrating a 23% incidence post-surgical myectomy.^4,11^

Although CAFs can be large, involve multiple branches and take complex courses, the majority are small, single, isolated lesions that are incidentally detected.^7^ They are often classified according to their site of origin, size, course, tortuosity and draining site, all key features that along with patient’s age, comorbidities, symptoms, haemodynamic consequences, and presence of associated complications (e.g. coronary artery aneurysms and thrombus) inform treatment strategies.^7^ The majority (80%) are reported to involve the right coronary artery, draining into right-sided cardiac structures with only 3–5% draining into the LV, although these data are not consistent.^7,10,12^

The clinical manifestations of CAFs vary based on the anatomical communication and the resulting haemodynamic consequences (including shunt properties). Clinical presentations range from asymptomatic and incidentally discovered CAFs, to angina due to coronary artery steal and myocardial ischaemia, heart failure resulting from progressive cardiac chamber dilatation and volume overload, atrial and ventricular tachyarrhythmias, and pulmonary hypertension.^7,8^ There is also a small but serious risk of coronary artery aneurysm formation and subsequent rupture.^7,8^

Transthoracic echocardiography is the commonest modality for the detection of CAFs, but its sensitivity is limited by available acoustic windows and is operator dependent. Diastolic flow on colour Doppler is typical for a coronary origin. Furthermore, echocardiography lacks the spatial resolution to confirm coronary anatomy, particularly in cases of tortuous arteries and small shunts.^13,14^ Invasive coronary angiography is considered the gold standard for diagnosis and evaluation of CAFs,^10,14^ although CTCA (especially with post-treatment technologies such as cinematic rendering) is considered more sensitive than invasive angiography for the detection of small fistulae and for evaluating the complex anatomical features (e.g. origin, course, drainage site, fistula number and diameter, and aneurysms) and associated coronary artery lesions (e.g. atherosclerotic plaques and myocardial bridges) necessary for surgical planning.^10,14^ Photon-counting CT will likely further increment the utility of cardiac CT. Currently, the role of cardiac magnetic resonance imaging in the evaluation of CAF is limited by low spatial resolution, long acquisition times and frequently encountered contraindications.^14^

Guidelines from the American College of Cardiology/American Heart Association recommend closure of CAFs that are large, symptomatic (i.e. myocardial ischaemia and arrhythmias), result in ventricular dilatation or dysfunction or are associated with coronary artery aneurysms (due to rupture risk).^7,8,10^ Closure may be performed via transcatheter intervention (i.e. coil/embolization) or surgical approach (via epicardial or endocardial ligation).^7,8,10,15,16^ Either treatment approach entails comparable low mortality risk (<1–2%), long-term durability, and low complication rates, with the choice of intervention largely dependent upon CAF anatomy and procedural risk.^7,8,10,15,16^ Patients with CAFs that are small and asymptomatic are usually treated conservatively with regular follow up including serial echocardiography every 3–5 years.

Whilst some congenital CAFs may close spontaneously, most acquired CAFs and especially coronary-cameral fistulae (our case) rarely close spontaneously.^7,10^ In contrast, high rates of spontaneous closure of small septal CAFs after surgical myectomy have been reported, depending on timing of echocardiography.^4,9,11^

Our case demonstrated several nuances in the evaluation of patients with CAFs. An individual approach to the assessment and evaluation of CAFs is required, however, multimodality imaging is vital to confirm anatomy and determine the need for intervention.

## Supplementary Material

ytae248_Supplementary_Data

## Data Availability

Data on this case report can be made available on application.

## Lead author biography

Dr James Bowles [Bachelor of Medicine/Bachelor of Surgery (BMBS), Fellow of the Royal Australian College of General Practitioners (FRACGP)] is a qualified General Practitioner currently undertaking Basic Physician Training with the Sunshine Coast Hospital and Health Service (Queensland, Australia). He is passionate about all aspects of Cardiology and wishes to pursue a career in Cardiology. He has previously trained at Gold Coast University Hospital (Queensland, Australia) after completing his medical degree at Deakin University (Victoria, Australia). Outside of work, he enjoys spending time with his wife at the beach and staying physically active.

## Supplementary material

**Consent**: The authors confirm that written informed consent has been obtained from the involved patient as per COPE guidelines. The patient has given approval for their information to be published in this case report.

**Funding:** Nil.
