# Supplementary figures and images for: Coronary artery fistula following surgical myectomy for hypertrophic obstructive cardiomyopathy: a case report
Source: Eur Heart J Case Rep. 2024 May 16;8(6):ytae248. doi: 10.1093/ehjcr/ytae248 (PMC11156195; doi:10.1093/ehjcr/ytae248)

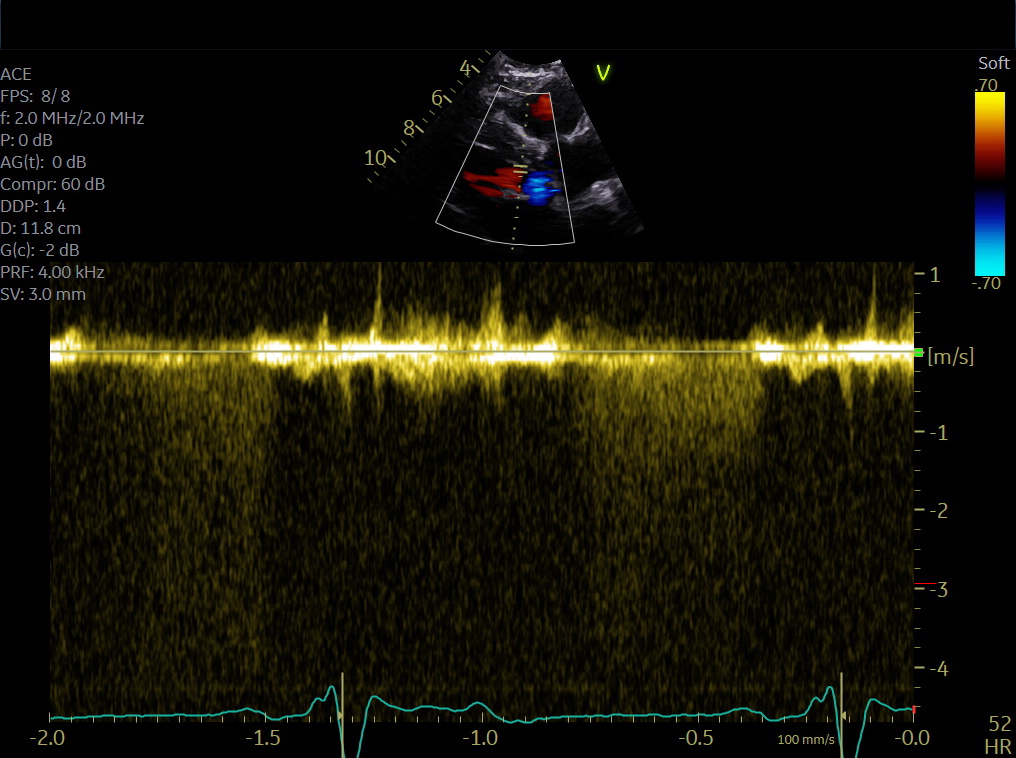

Supplement: ytae248_Supplementary_Data [file ytae248_supplementary_data.zip › Figure S1 - PW Doppler CMYK.tiff]

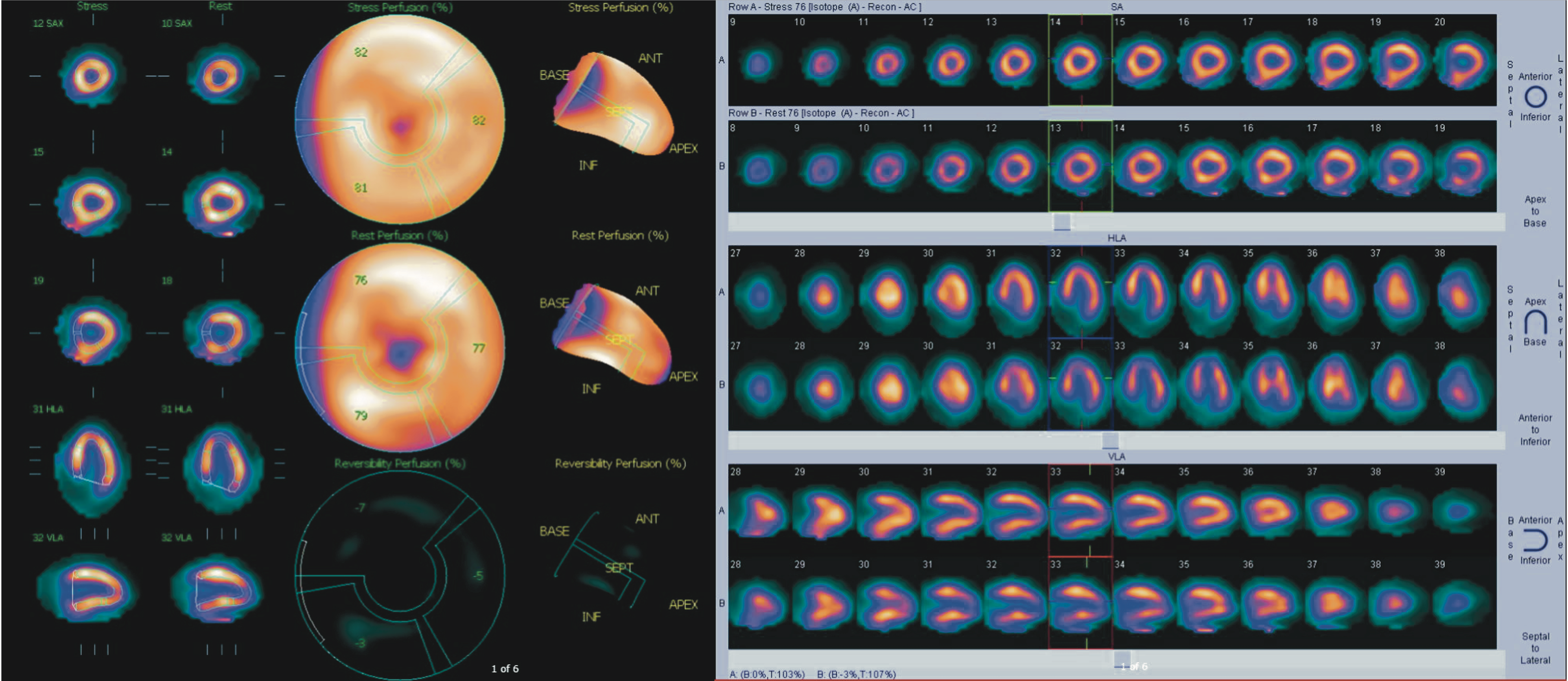

Supplement: ytae248_Supplementary_Data [file ytae248_supplementary_data.zip › Supp2.tiff]
